# How to get back on track? Experiences of patients and healthcare professionals regarding weight recurrence and needs for an intervention after bariatric-metabolic surgery

**DOI:** 10.1016/j.obpill.2023.100074

**Published:** 2023-06-08

**Authors:** Vera Voorwinde, Sahar Moukadem, Maartje M. van Stralen, Ignace M.C. Janssen, Valerie M. Monpellier, Ingrid H.M. Steenhuis

**Affiliations:** a Nederlandse Obesitas Kliniek (Dutch Obesity Clinic), Huis ter Heide, the Netherlands; b VU University, Faculty of Science, Department of Health Sciences and Amsterdam Public Health Research Institute, Amsterdam, the Netherlands

**Keywords:** Bariatric-metabolic surgery, Behavior modification, Lifestyle intervention, Patient experience, Weight recurrence

## Abstract

**Background:**

Multidisciplinary lifestyle interventions are recommended as a first step in treating weight recurrence after bariatric-metabolic surgery (BMS). However, little is known about the experience of patients and healthcare professionals (HCP) with these interventions and how they should be tailored to the patients’ needs. The aim of this study was to gain more insight into the experiences and needs of patients and HCP regarding weight recurrence after BMS and an intervention to get Back on Track. In addition, attitudes towards integrating e-Health into the care program were explored.

**Methods:**

A qualitative process evaluation of an intervention for weight recurrence, the Back on Track (BoT), was conducted by means of in-depth interviews and focus groups with 19 stakeholders, including patients and HCP involved in BoT. Interviews were transcribed verbatim. Data were analyzed through thematic analysis.

**Results:**

Patients and HCP reported a wide array of causes of weight recurrence. Patients found it difficult to decide when weight recurrence is problematic and when they should ask for help. Patients reported feeling like the exception and ashamed, therefore experiencing a high threshold to seek help. E-Health was seen as a promising way to improve tailoring, screening, autonomy for the patient, and accessible contact.

**Conclusion:**

Patients should be adequately counselled on weight recurrence after BMS and the importance of intervening early. It is important to lower the threshold for seeking help. For example by offering more long-term standard care or by adding e-Health to the intervention.

## 1 Introduction

Weight recurrence is a major long-term problem for an estimated 20–30% of patients who have undergone bariatric-metabolic surgery (BMS) [[1], [2], [3]]. Weight recurrence can result in recurrence or deterioration of obesity-related medical problems and is associated with deterioration of Health-Related Quality of Life (HRQoL) [[4], [5], [6], [7]]. Optimization of long-term care is therefore important to prevent or adequately treat weight recurrence, when necessary.

A wide range of risk factors have been identified that may contribute to weight recurrence. These include anatomical factors such as dilation of the gastric pouch, changes in hormones such as ghrelin and glucagon-like-peptide-1 (GLP-1), genetics, as well as different behavioral and psychological factors [8,9]. Behavioral and psychological factors that are believed to be critical to long-term weight recurrence include increased food urges and caloric intake, increased portion size, emotional eating, loss of control when eating, anxiety, and several psychiatric conditions [[8], [9], [10], [11], [12]].

Given the multifactorial causes, a multidisciplinary approach to the management of weight recurrence, tailored to the needs of the patient, is essential [8,9,13,14]. Although for some patients surgical revision or pharmacological treatment may be the best option, for the majority of patients, early multidisciplinary lifestyle interventions to stop or prevent further weight recurrence should be the first step [13,14]. What exactly these interventions should entail and how they should be adjusted to the patients’ needs, is still unclear. Moreover, to be able to tailor these interventions to the needs of the patient, insight into the long-term experiences and needs regarding weight recurrence after BMS and an intervention to get Back on Track is critical.

Only a few studies have focused on experiences with long-term care. They show lasting challenges in maintaining a healthy lifestyle, as well as difficulties with developing new coping strategies, and with finding the right support [[15], [16], [17]]. None of the studies included other stakeholders, such as healthcare professionals (HCP). In addition, a large proportion of patients does not seem to benefit from the long-term care programs, given the generally very low attendance [[17], [18], [19]]. Greater understanding of patient needs regarding long-term care may also improve engagement and attrition to future interventions. Barriers to attend follow-up care include shame, time, cost and travel distance [[18], [19], [20]]. The use of e-Health may tackle some barriers to attend follow-up care, facilitating more accessible support [18,21,22]. Some studies showed promising results using e-Health postoperatively and for weight recurrence after BMS specifically [21,23].

Hence, the aim of this study was to gain more insight into the experiences of patients and HCP with weight recurrence after BMS and an intervention to get Back on Track. A qualitative process evaluation of the current intervention for weight recurrence was conducted via in-depth interviews with patients and HCP. In addition, attitudes towards integrating e-Health into the intervention were explored. The results can inform intervention development, and increase engagement of patients with these interventions.

## 2 Methods

### 2.1 Setting

This study was conducted at the Dutch Obesity Clinic (Nederlandse Obesitas Kliniek (NOK), which is a multicenter outpatient clinic providing pre- and postoperative care for patients undergoing BMS. The NOK offers a comprehensive interdisciplinary pre- and postoperative treatment program until 12 months after surgery [24]. After that, patients enroll in the follow-up program wherein they return to the clinic every year for a follow-up meeting. When weight recurrence or relapse is signaled, either by the patient itself or by the physician, the Back on Track (BoT) intervention is offered. There are no strict guidelines for the amount of weight recurrence to enter the intervention, it is advised that patients with weight recurrence of more than 10% in a short period or more than 15% in a number of years to follow the BoT.

The aim of the BoT intervention is weight stabilization/weight recurrence prevention by counselling sessions focusing on getting all relevant lifestyle behaviors “back on track”. These behaviors include lifestyle in a broad sense and include dietary behavior and physical activity, as well as factors such as stress, sleep, emotion regulation and planning skills. Consultations follow the guidelines of motivational interviewing [25]. The BoT consists of 3 counselling sessions with a member of the interdisciplinary treatment team. Depending on the needs of the patient, consultations will be scheduled with 1, 2 or all 3 of the practitioners (i.e., dietitian, psychologist or physical therapist) in subsequent consults. The intervention is completed with an evaluation by the physician/nurse specialist. The intervention is planned over a period of 4 months.

### 2.2 Study design

A qualitative process evaluation was conducted through semi-structured interviews with patients who participated in the BoT, and focus groups with interdisciplinary teams of HCP who are involved in the BoT. This study was conducted according to the ethical standards declared in the Declaration of Helsinki. Following the criteria of the Dutch Medical Research Involving Human Subjects Act, our study complies with the Code of Ethics of the Faculty of Science of the Vrije Universiteit Amsterdam; therefore, our study did not require further evaluation by the Research Ethics Review Committee.

### 2.3 Participants

Participants were recruited at 4 different locations of the NOK. Patients were eligible to participate in this study when they had finished the BoT less than 3 months prior to the study. A purposive sampling technique was used to select a mix of patients that reflects the heterogeneity of the population. Patients were contacted by a member of their own treatment team, who asked if they were willing to participate in this study. HCP were recruited at 3 different locations and contacted by the researcher directly. Written informed consent was obtained in all participants before the start of the interview or focus group.

### 2.4 Data collection and analysis

Data collection was deemed saturated and showed sufficient depth to meet the aims of the study, with a total of 19 participants; 8 interviews with patients and 3 focus groups with a total of 11 HCP. After 6 interviews, the information from the analysis did not lead to any changes in the codebook. The research team collectively decided that 2 more interviews, including one with a male participant, were needed to reach data saturation. Five interviews were conducted in the patients’ homes, and 3 at a NOK location, depending on the preference of the patient. All focus groups were held at a NOK location. Focus groups consisted of the BoT team at the location. Interviews lasted approximately 1 h, focus groups approximately 90 min. The interview guide was developed by the research team and was based on a theoretical framework derived from relevant literature concerning weight recurrence and process evaluation topics.

All interviews and focus groups were conducted by the first author (VV) who is a PhD student and also works as a psychologist at one NOK location. She has experience in pre- and postoperative care for bariatric patients. VV had no treatment relation with participating patients, she introduced herself as researcher, and patients were informed about the goals of the study in an information letter provided before obtaining informed consent. Interviews and focus groups were audio-recorded, de-identified, and transcribed verbatim.

The data were analyzed through thematic analysis following the 6 main stages [26]. Inductive and deductive techniques were combined to base the data on the theoretical framework while also allowing themes to emerge from the data [27]. MaxQda software packages were used for coding. Transcripts were independently coded by the first (VV) and second (SM) author. The second author (SM) finished her bachelor health sciences and was trained in conducting qualitative research. VV and SM both conducted the same analyses of all interviews and focus groups, after which they jointly reviewed and discussed all codes to reach agreement. The resulting code tree was discussed in the research team.

## 3 Results

### 3.1 Participant characteristics

Most patients underwent Roux-en-Y Gastric Bypass (RYGB) 3–5 years before study start. The 3 focus groups consisted of interdisciplinary teams of HCP who were all involved in and experienced with the BoT program. The participant characteristics are described in Table 1.

**Table 1:** Characteristics of the study participants, the patients (n = 8) and interdisciplinary teams (n = 11).

Patient	Age	Gender	Years since surgery	Surgery Type	Nadir Weight (BMI)	%TWL	Weight at start BOT (BMI)	%TWL	Weight after BoT (BMI)	%TWL
P1	51	F	4	Redo	84.7 (34.8)	17	92.3 (37.9)	9	93.7 (38.5)	8
P2	68	F	4	RYGB	78.4 (33.8)	29	89.1 (30.5)	19	87.2 (29.8)	21
P3	44	F	6	RYGB	74.6 (28.4)	28	79.4 (30.3)	23	77.5 (29.5)	25
P4	31	F	5	RYGB	83.4 (26.3)	24	98.2 (31)	10	95.1 (30)	12
P5	63	F	3	RYGB	93.6 (34.8)	25	111 (41.3)	11	117.4 (43)	6
P6	46	F	5	RYGB	77.5 (28.8)	30	89 (33.1)	20	91.2 (33.9)	18
P7	54	M	4	RYGB	115.7 (38.2)	30	133.6 (44.1)	20	133.8 (44.2)	19
P8	41	F	5	RYGB	74.6 (27.4)	37	91.3 (33.5)	23	83.9 (30.8)	29
**Team**	**N**	**Gender**	**Discipline**							
T1	4	2F, 2 M	1 Dietician, 1 Nurse specialist, 1 Physical Therapist, 1 Psychologist							
T2	2	2F	1 Physical Therapist, 1 Psychologist							
T3	5	5F	1 Dietician, 1 Physician, 1 Physical Therapist, 1 Psychologist, 1 Care coordinator							

TWL = Total Weight Loss, BMI= Body Mass Index, BoT = Back on Track, F = female, M = male, Redo = Conversion from Laparoscopic Adjustable Gastric Band to Roux-en-Y Gastric Bypass, RYGB = Roux-en-Y Gastric Bypass.

### 3.2 Themes

Six main themes were found. All themes and subthemes can be found in Table 2.

**Table 2:** All themes with subthemes expressed by patients and interdisciplinary treatment teams.

**1. Weight recurrence can happen to anyone**
**1.1 Different trajectories**
• Of weight loss in the initial phase after surgery
• The turning point/timing of weight recurrence
**1.2 Different causes of weight recurrence**
• Physical
• Dietary behavior
• Psychological
• (Social) environment
• (physical) activity
**2. When to raise the alarm?**
• Timing of the intervention, often too late
• Head-in-the-sand,
**3. Barriers for seeking help**
• Unaware of existing help
• Perception of being the exception
• Shame
**4. Experiences with the intervention**
• Valued long-term support
• Different results
• Higher expectations, less satisfaction
• Autonomy vs. Guidance
**5. Suggestions for improvement**
• Support groups
• Back on Track part of standard treatment
• Better promotion and earlier intervention
• Structure and clarity
• Tailoring
**6. Perspectives on eHealth**
• Intention to use
• Pros
• Cons
• Suggestions

#### 3.2.1 Theme 1: weight recurrence can happen to anyone

##### 3.2.1.1 Different trajectories

In the initial phase after surgery, most patients could not yet suspect that weight recurrence would occur in the longer term. Patient experiences were quite different from each other. Most patients reported everything went really well from the start, others reported losing weight too fast in the beginning. While a single patient started to experience weight recurrence from early on in the trajectory, for most of them it started after about two years after surgery.

*After two or three years, it gets harder. In the beginning it all went well, but after … the weight gain, I found that really difficult. P2*

##### 3.2.1.2 Different causes

Patients described the feeling that, from about 2 to 3 years after surgery, their stomach had become larger. They felt they could eat larger amounts of food and their appetite increased.

*Yes, I can eat much more, way more than in the beginning, I think my stomach has gotten bigger. P1*

However, they also described that the cause of their weight gain lied deeper than that. They mentioned many different causes, in different domains ranging from physical changes, social environmental, psychological, and (dietary) behavioral causes.

*Support is very important, but the stress has also caused emotional eating. P6*

As part of the social environment domain patients mentioned feeling abandoned from the support of the clinic. During the first year after surgery, there are regular visits to the clinic and meetings with their support group that change into the follow-up phase with just one appointment a year.

*It's about the check-ups, you know? You have the meetings; you get the brochure … this is what you have to do … so it stays fresh in your mind. But at a certain point you are left on your own, P2.*

The lack of support from family members at home was also mentioned as a contributing factor to the weight gain.

In the domain of dietary behavior, patients mentioned that their knowledge had subsided over the years. Old habits, such as dieting, returned as well as urges and cravings for unhealthy food. This led to no longer following the nutritional guidelines.

At the root cause of weight recurrence, however, many patients described a psychological cause. Almost every patient mentioned some kind of major life event, stress, poor self-care, low self-confidence, emotional eating and even depression as the starting point or underlying cause of the changes in the other lifestyle factors. As a result, exercise was no longer a priority, patients lost structure, and stopped monitoring their weight and lifestyle behaviors.

*I have the family situation so to speak, our child has behavioral problems so there was just a lot of stress and I threw myself completely onto my work. So, it was also stress-related, I was very tired and I am an emotional eater. When I am stressed I eat the wrong things, P8.*

The HCP also mentioned various different causes and noticed it often started with lifestyle imbalance. Patients get out of balance because of some incident or major life event which is mostly negative, but could also be positive such as changing jobs, having a baby, or moving to a different place. After which it becomes very difficult to maintain a healthy lifestyle on many different levels.

#### 3.2.2 Theme 2: when to raise the alarm?

An important question that was asked especially by patients was: when do you raise the alarm? Recognizing the difference between normal weight gain that is part of weight stabilization and problematic weight recurrence that should be treated proofed to be difficult for patients.

*You are told that you will gain some weight, but what is normal and when do you raise the alarm? P1.*

HCP also observed that the patients themselves very rarely asked for help. Weight recurrence was usually discussed during the follow-up visits, and confirmation (or confrontation) from a physician was needed.

*I don't think patients ask for the BoT themselves. They attend the follow-up and we advise them to follow the BoT. T1.*

Some patients said they buried their heads in the sand and that it was only by talking to the physician that they really realized how bad it was.

*I knew it was coming, but then it was still easier if someone else said it to me than if I said it myself. P6.*

As a result, patients often received help later than the patients and HCP ideally wanted.

#### 3.2.3 Theme 3: barriers for seeking help

A barrier to seek help frequently mentioned by both patients and HCP is shame. Both HCP and patients also mention patients often feel like they are the only ones who experience weight recurrence.

*Patients say they feel like a failure, that they went through surgery and the whole trajectory, and now have to return for help again. T2.*

In addition, HCP fear that the patients who need it most are most ashamed, don't attend the follow-up, and therefore will not be reached at all. Patients mostly indicate that they want to solve their problems themselves, and were therefore reluctant to seek help.

#### 3.2.4 Theme 4: experiences with the intervention

Both patients and interdisciplinary teams appreciated the fact that the BoT is offered. Interdisciplinary teams called it a moral obligation to offer long-term care.

*You have to offer it. We support and coach patients with a chronic disease, we can't say we only help you from then up until then, and after that you are on your own. T1.*

The intervention seemed sufficient for people who just needed a little push to get their lifestyle back on track. However, patients expect and hope to lose weight during the BoT. HCP noticed that patients with higher expectations, hoping to lose a lot of weight, were less satisfied with the BoT. As a result, they were often practicing expectation management.

*I also think that in terms of weight loss they expect more, and in terms of behavior change we expect more than is happening. T3.*

If patients could choose a HCP they mostly went to the dietician, even though this did not always seem to be the best fit for their problems. Some patients appreciated that they could choose, others said they could have used more guidance.

*And of course, I was asked: do you want to see a psychologist? No, of course I don't. She will stir emotion; I don't want that. so that's very easy. So maybe yes, a little more structure. P6.*

Interdisciplinary teams admit they may also have referred to the dietician to start with, too easily sometimes. They believe that if they had more time, they might have found out that the underlying cause of the problems with the diet was in fact psychological.

#### 3.2.5 Theme 5: suggestions for improvement

Patients suggested extending the standard treatment program. They would prefer a mix of individual and group treatment. Patients reported missing group support like they had in the beginning after surgery.

*Because I think it's a necessity for everyone to come back again in those 5 years. Not just for the scale [weight], but just a refresher course. P1.*

Furthermore, if the BoT were part of the standard treatment program, patients think they would feel less abandoned, less like exceptions and perhaps less ashamed.

*Contact with other patients who experience the same. I'm the only one in my own group who put on weight. It would be nice to share experiences. P8.*

Interdisciplinary teams also noticed that patients miss group support and think a group can be a good addition to the BoT intervention. They worry though, that turnout to these group sessions will be low. They also worry about group safety when they only meet a couple of times. They reported better promotion, also for follow-up appointments, is needed.

Patients would have liked to have more consultations, or at least more flexibility to have had a couple more when needed. They want their needs to be listened to*.* If the intervention is not appropriate to treat their problems, then they hope for help in finding treatment options externally. Like patients, teams also think it would be better to be able to schedule the BoT more flexibly. A suggestion was to bring forward the evaluation with the physician, and to use this evaluation to determine whether, and if so, how much, treatment is still needed.

Patients reported they are looking for something to hold on to. They report they would have preferred a list or menu for nutritional guidelines. Also, they ask for clear communication about the structure and content of the intervention, and they think it is important that information and advice are consistent across different practitioners. Some patients prefer to see the same practitioner for the whole BoT intervention, while others prefer to see all four different disciplines.

#### 3.2.6 Theme 6: perspectives on e-health

Interdisciplinary teams reported they liked the concept, but they worried patients will not use e-Health. To be able to use it themselves, the teams reported it is important to be able to follow the patient's progress via a practitioner's portal. They mentioned seeing advantages in tailoring, possibilities for screening, more autonomy for the patient, and more accessible contact.

*In any case, this would be a good development in several respects, you can focus your conversation because you already have information, you have an information flow from app or laptop materials completed, cost-benefit technically that is also much more efficient because you are much more focused, so the expected effect is greater. T1.*

A number of patients indicated that they had asked for an app years ago. All reliable information in one place, is what they would like. One patient hardly uses apps in daily life and would not find them useful for this purpose either.

*It's not personal, I liked the face-to-face appointments. P7.*

Both HCP and patients suggested the app should contain little text, should be clear, and easy to use. It should be easy to look up and retrieve information. Positive reinforcement is important as well. With regard to content they suggested, among other things: a functional food diary with feedback on request, recipes, information about dealing with difficult situations, sports tips with sample video's, information videos, and tips for remaining focused. A few patients mentioned it would be helpful if the app could signal relapse in the future.

## 4 Discussion

In this study we found that weight recurrence can happen to anyone, and patients often don't know when to ask for help. Moreover, shame is an important barrier to ask for help. Both patients and HCP appreciated the availability of an intervention for weight recurrence, patients even suggested this intervention should be a standard part of the treatment program after BMS. E-Health might be a helpful addition to the program.

An important finding is that it is largely unclear to the patient when exactly their weight recurrence is a problem, and thus when they need to ask for help [28]. This is understandable since a clear definition of weight recurrence is also still lacking among clinicians and researchers [3,6]. Several researchers and expert groups have looked at the question of when weight recurrence is clinically relevant. Although there is no standardized definition, there seems to be agreement that weight recurrence relative to nadir weight or weight recurrence relative to total weight lost would be most appropriate, as both produce the least bias when comparing patients with different preoperative BMI, and show the strongest associations with clinical outcomes [3,6,29,30]. A survey among surgeons also revealed that weight recurrence relative to nadir weight was preferred by the majority of the surgeons [31]. A staging system to classify different stages of weight recurrence seems accurate as opposed to a dichotomous measure, because of the dose-response relationship of weight recurrence with the return of obesity-related medical problems and decline in HRQoL [6,29]. We endorse the Post Operative Weight Recurrence (POWER) taskforce's proposal for a consensus process to arrive at a suitable definition [29]. Our study shows that in addition to the relevant expert groups, the patient perspective is very important in the consensus process. A definition of weight recurrence should be understandable and meaningful to the patient. In the meantime, it is important to encourage patients to monitor their weight gain and to counsel patients before surgery that weight recurrence is not a personal failure but a medical problem that needs multidisciplinary assessment and treatment.

The wide array of causes of weight recurrence cited by both patients and professionals also demonstrates the need for treatment by a multidisciplinary team [32]. Patients' and HCP’ perspectives on the underlying causes of weight recurrence, largely corresponded with the existing literature on weight loss maintenance and also with literature on relapse in different domains such as addiction [28,32]. The causes, which were mainly psychological, indicate that there is a particular need for treatment by a psychologist. This was also mentioned by both HCP and patients. This is opposite to the current back on track procedure but in agreement with previous research that reported that, although not specifically for weight recurrence, patients mention psychological support as one of the most important components of care [33]. The experience that weight recurrence often started with some major life event or lifestyle imbalance that caused changes in the other lifestyle factors, corresponds with Marlatt's model on relapse [34] and a review on weight loss maintenance [35]. According to Marlatt's model, lifestyle imbalance can increase higher desire for indulgence, and consequently the probability of a lapse (i.e., slip) in a healthy behavior arises when an individual enters a high-risk situation, i.e. any situation that poses a threat to the individual's sense of control, without effective coping skills [36]. Effective strategies to prevent lapse and consequently weight recurrence could be to improve lifestyle balance by identifying stressors, and acquiring sufficient stress management strategies by for example balancing ‘shoulds’ (i.e. external demands) in an individual's life with ‘wants’ (i.e. activities that give energy/are enjoyable). Also, identifying high risk situations and acquiring effective coping skills while entering the high-risk situation could be effective strategies for a back on track intervention [34].

The topics revealed particularly striking results in the areas of reach and recruitment. It is known that people who experience weight recurrence often blame themselves and are therefore less likely to contact HCP for help [32,37]. Also, in the current study patients encountered several barriers for seeking help, of which shame was a very important one [28,37]. They feel alone, and pro-active support by HCP instead of patients needing to initiate contact, is perceived as very helpful [17,33,37]. This was also reflected in the current study where patients suggested offering this intervention to all people who had undergone BMS. Moreover, patients suggested help in groups, which will aid with the feelings of loneliness and might improve outcomes [33,37]. In addition, patients often don't expect the weight recurrence, thus it is crucial to educate patients that obesity is a chronic, relapsing disease that requires lifelong treatment and that patients who need long-term care are therefore not an exception [37].

As advised in current guidelines, both patients and HCP value long-term care and deem it necessary after BMS [38]. Though they both mentioned that better promotion is important. According to social marketing principles, it is essential that the intervention is promoted when it is most desired in a way that is most easily understood [39]. Patients also expressed their needs for clear communication, structure and guidance towards appropriate care. They were often looking for something to hold on to and stressed the importance that information and advice are consistent across different HCP. Thus, it seems that the availability of the BoT intervention should be mentioned more throughout the treatment program, preferably starting at 12 months after BMS. This moment can then also be used to address the fact that weight recurrence is a common problem. Also, following the social marketing strategies, when addressing this, the BoT intervention should then also be offered as standard care (the default option), instead of an optional treatment.

Both patients and HCP view e-Health as a promising development to improve care. Patients and HCP provided numerous ideas to implement in e-Health interventions. Intervention developers should consider these ideas to increase engagement, whilst taking evidence on key elements that promote behavior change such as the behavior change taxonomy or intervention mapping into account [40,41]. Furthermore, important disadvantages of e-Health should be considered. Patients mentioned it is less personal, and expressed concerns with less digital literate patients; hence blended care, wherein e-Health is combined with the face to face (group) treatment with a HCP, seems to be more fitting to maintain the personal contact.

There are several strengths and limitations to this study. A strength is that multiple stakeholders in long-term care were part of the study. Interviews were coded by 2 researchers independently. A limitation is that we only interviewed patients who finished the intervention, therefore the perspective of patients quitting the intervention was not taken into account. This would be interesting for future research.

## 5 Conclusion

This study provides insights into the experiences of patients and HCP with weight recurrence and an intervention for weight recurrence with several implications for long-term care. First, long-term care should be more accessible or incorporated in standard care when possible, to prevent feelings of shame and feeling an exception. Second, to tailor the intervention to the patient's needs it is important to start the intervention with a thorough assessment of all factors influencing weight recurrence, including lifestyle imbalance and stress, in order to identify focal points for treatment. Third, patients benefit from structure and clarity and need to retrieve all reliable information in one place. E-Health in addition to the regular intervention is a promising way to improve tailoring, possibilities for screening, more autonomy for the patient, and more accessible contact.

## Author contribution

All authors made substantial contributions to the conception and design of the submission. Data were collected by VV. Analysis and Data Curation was performed by VV and SM with additional input from MM, VM and IS. VV wrote the first draft. All authors reviewed, edited, and approved the final submission for publication.

## Ethical review

This study was conducted according to the ethical standards declared in the Declaration of Helsinki. Following the criteria of the Dutch Medical Research Involving Human Subjects Act, this study complies with the Code of Ethics of the Faculty of Science of the Vrije Universiteit Amsterdam. Written informed consent was obtained in all participants before the start of the interview or focus group.

## Source of funding

This manuscript is not funded by any specific grant from funding agencies in the public, commercial, or not-for-profit sectors.

## Declaration of competing interest

The authors declare the following financial interests/personal relationships which may be considered as potential competing interests: V. Voorwinde reports financial support was provided by De Nederlandse Obesitas Kliniek BV. V.M. Monpellier reports financial support was provided by De Nederlandse Obesitas Kliniek BV. I.M.C. Janssen reports financial support was provided by De Nederlandse Obesitas Kliniek BV. V. Voorwinde reports a relationship with De Nederlandse Obesitas Kliniek BV that includes: employment. V.M. Monpellier reports a relationship with De Nederlandse Obesitas Kliniek BV that includes: employment. I.M.C. Janssen reports a relationship with De Nederlandse Obesitas Kliniek BV that includes: board membership and consulting or advisory.
